# Cause Analysis of Unsafe Behaviors in Hazardous Chemical Accidents: Combined with HFACs and Bayesian Network

**DOI:** 10.3390/ijerph17010011

**Published:** 2019-12-18

**Authors:** Xiaowei Li, Tiezhong Liu, Yongkui Liu

**Affiliations:** 1 School of Management and Economics, Beijing Institute of Technology, Beijing 100081, China; bitlxw@163.com; 2 Macro Strategy Research Office, Chinese Academy of Labour and Social Security, Beijing 100029, China; liuyongkui@calss.net.cn

**Keywords:** hazardous chemical accidents, human factors analysis and classification system, Bayesian network, operation error, operation violate

## Abstract

Hazardous chemical accidents (HCAs) seriously endanger public life, property, and health. Human and organizational factors are important causes of many kinds of accidents. In order to systematically explore the influencing factors of unsafe behaviors in HCAs in China, the method of human factors analysis and classification system based on the Bayesian network (BN-HFACs) was introduced. According to the 39 investigation reports of HCAs in China, the origin Bayesian network (BN) was obtained and the failure sensitivity of every node in BN was calculated. The results have shown that hazardous material environment (1.63) and mechanical equipment (0.49) in the level of preconditions of unsafe behavior have the same direction failure effect with operation error, while there is no factor has the same direction failure effect with operation violate. Some factors in organization influence and unsafe supervision, such as organization climate (0.34), operation guidance (0.37), planned operation (0.22), and legal supervision (0.19), are also important reasons for operational errors, while resource management (0.12), hidden investigation (0.18) and legal supervision (0.13) have an impact on operation violates. Moreover, there are still close relationships between other hierarchical elements, such as the operation guidance effect on the hazardous material environment (6.60), and the organizational climate has the most obvious impact on other factors at the level of organizational factors. Based on the above research conclusions, suggestions for individual, enterprise, and government were put forward, respectively, and the limitations of this study were also clarified.

## 1. Introduction

With the worldwide economy rapidly developing, there is an increasing demand for the production and usage of hazardous chemicals. However, hazardous chemicals also bring a huge threat to people’s security of life, property, health, living environment, etc. Although the number of hazardous chemical accidents (HCAs) in China has shown a downward trend in recent years [1], there are still a large number of casualties and economic losses caused by HCAs. Based on the analysis of 3974 HCAs in China from 2006 to 2018, it has been found that 50.57% of HCAs were caused by unsafe behavior [2]. Similarly, it has been found that 35% of all HCAs in China from 2007 to 2010 were caused by the violation of operation rules [3], and 44% of all HCAs in China from 2011 to 2013 were caused by unsafe behavior [4]. Furthermore, according to the analysis of 27 typical HCAs in 2016, the unsafe behavior of operators is a main cause [5]. It could be seen that human unsafe behavior is the main factor of HCAs. Therefore, in order to reduce the occurrence of HCAs, it is necessary to study the unsafe behavior and the influence factors of unsafe behavior in the context of HCAs.

There are various models has been proposed to study the causation of the accident from the view of human unsafe behavior or human errors, including Accident Prone Tendency (APT), Accident Liability (AL), Surry’s model, Hale’s model, Wigglesworth’s model, Lawrence’s model, and so on. Among them, APT and AL are relatively vague about human factors in an accident, while the other four models are accident analysis models based on a human cognitive process that are suitable for analyzing human errors in accidents. However, the above models did not discuss the organizational factors in detail [6]. Since then, in 1990, Reason proposed the Swiss Cheese Model (SCM) [7], which fully considered the organizational factors. According to SCM, accident causation could be divided into four levels, including organizational factors, unsafe supervision, preconditions for unsafe conditions, and unsafe actions. However, the SCM did not subdivide the four levels of specific indicators. In 2001, based on the SCM and the flight data of the U.S. military and civil aviation, Shappell and Wiegmann [8] proposed the Human Factors Analysis and Classification System (HFACs). Compared with SCM, HFACs specified the four levels of accident causation.

Due to the clear logic and convenient application of HFACs, the model has been widely used in recent years. Chen et al. used HFACs to study the cause of ship collision accidents, and found that human and organizational factors have more significant impact on the occurrence probability [9], while Zhang et al. used HFACs to investigate the contributing factors of major road traffic accidents and found that unsafe acts, violations, and inadequate regulations are the most important factors [10]. Similar researches also exist in various fields, such as maritime [11,12,13], coal mine [14,15,16], aviation [17,18,19], transportation [20,21,22], construction [23,24], and so on. Hulme et al. [25] collected 43 studies, which analyzed the causes of accidents by HFACs from 2000 to 2018 and found there are 15 studies belong to the field of aviation, and there are 10, seven, six, and two studies belong to the field of maritime, mining, transportation, and construction, respectively. The other three studies focused on the nuclear and oil and gas industry. Kim et al. [26] used HFACs to investigate the relationship between all of the hazards from the view of organizational factors in the field of nuclear industry. Yoona et al. [27] proposed a new method for modeling and analyzing human-related accidents, which integrates HFACs and activity theory-based approach; the method was verified by a nuclear plant case. Theophilus et al. [28] used HFACs to analyze the organizational factors and human errors in the oil and gas industry.

Based on the above research review, it can be found that HFACS has been widely used in many fields to explore the organizational and human factors of accidents. However, in general, there are few kinds of research in the field of hazardous chemical accidents. In fact, with the outbreak of the dangerous chemicals accident in Tianjin, China on 12 August 2015, Chinese scholars pay more and more attention to the human causes of hazardous chemical accidents, and relevant researches have gradually emerged. Zhou et al. [29] established the modified HFACS model under the situation of the hazardous chemicals to analyze the human factors of hazardous chemical accidents in China and then analyzed the Tianjin “8.12” accident as a case. Zhang et al. [30] compared HFACs, AcciMap, and STAMP with the Tianjin “8.12” accident, and thought HFACS was a suitable method for practical application. Jiang and Han [31] used fault tree analysis and HFACs to systematically analyze the human factors of the guanidine nitrate explosion accident that occurred at HEBEI KEEPER Chemical Industries Co., Ltd. (Zhangjiakou City, China). It could be found that most of the existing research on the analysis of the human factors of dangerous chemical accidents using HFACS is based on a single case of dangerous chemical accidents, and there is a lack of general research on the human factors causation in dangerous chemical accidents.

Identifying the general causes of hazardous chemical accidents is conducive to enterprises and government departments to take more effective measures to reduce the occurrence of hazardous chemical accidents. Therefore, this study will take many accidents in China in recent years as examples, and use HFACs model to analyze the general causes that lead to hazardous chemical accidents. In the present article, a revised HFACs for HCAs is proposed, and 39 cases of HCAs from 2010 to 2018 of China are collected to support this study. In order to better analyze the relationship between accident causes from the quantitative perspective, the Bayesian network (BN), which was widely used in accident analysis and prediction, is introduced. The method of combining HFACs with BN, which also named BN-HFACs, has been used by many scholars and proved to be an effective method [32,33,34,35]. In sum, this study will use the BN-HFACs method to analyze the 39 cases of HCAs of China, in order to get the general causes of hazardous chemical accidents and provide theoretical support for reducing the incidence of hazardous chemical accidents in China and even in the world.

## 2. Materials and Methods

### 2.1. Revised HFACs for HCAs

**Figure 1:**
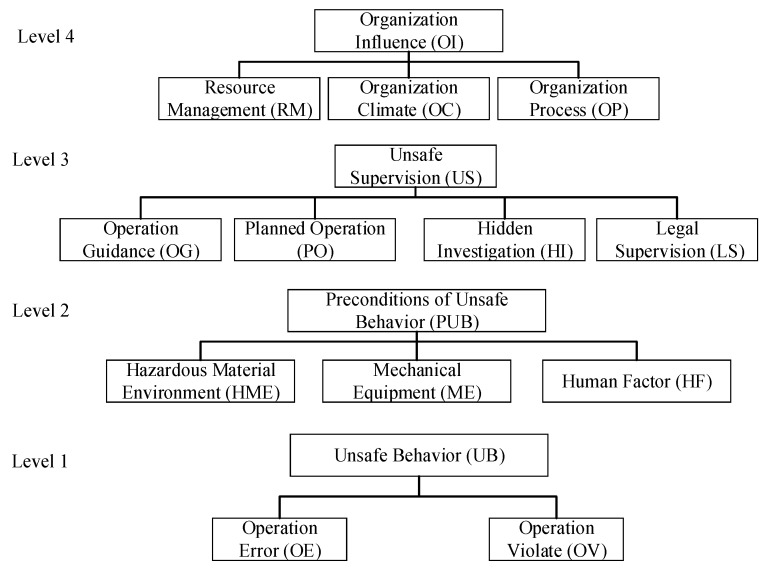
Revised human factors and classification systems (HFACs) for hazardous chemical accidents (HCAs).

Shappell and Wiegmann [8], who are experts of the Federal Aviation Administration (FAA) in the U.S., proposed HFACs to analyze the human error in aviation accidents based on the Swiss Cheese Model (SCM) [7]. The accident causation chain of HFACs is that “accidents were caused by dominant causes, and the dominant causes led by potential causes”. In the above chain, the dominant causes refer to human unsafe behavior, and the potential causes include the preconditions of unsafe behavior, unsafe supervision, and organizational influence. Thus, the accident causation chain also could be donated as “organization influence → unsafe supervision → preconditions of unsafe behavior → unsafe behavior”. However, the HFACs was initially proposed based on aircraft accidents; thus, in order to analyze the HCAs, the typical HFACs need to be revised based on the characteristics of HCAs. Figure 1 shows the revised HFACs for HCAs, and the specific definitions of each subdivision index are shown in Table 1.

### 2.2. Bayesian Network (BN)

Bayesian network, also known as belief network or causal network, is a probabilistic network composed of the directed acyclic graph (DAG) and conditional probability table (CPT) [36]. There are two steps to construct BN. First, the topological relationship between random variables should be determined. Second, CPT construction should be completed.

DAG is donated by G=〈V,E〉, the elements in V represent variables, and the directed edges E represent the relationships among variables. In DAG, if there is a directed edge pointed by the node $X_{1}$ to the node $X_{2}$, then, the $X_{1}$ is the parent node of $X_{2}$, and the $X_{2}$ is the child node of $X_{1}$. If there is a node that has no parent, then it can be called a root node; moreover, one node can be called a leaf node if it has no child.

CPT is used to represent the conditional probability distribution of nodes in DAG when nodes are discrete random variables. Every node in a DAG has a conditional probability distribution, and the conditional probability is defined as in Equation (1) according to the Bayesian formula”

(1) $$P(A|B)=\frac{P(A)P(B|A)}{P(B)}$$

where P(A) represents prior probability and P(A|B) represents posterior probability.

While only prior probability needs to be calculated for the root nodes, for the nodes with parent nodes, its joint conditional probability for all the parent nodes needs to be calculated according to the Bayesian formula. There exists a property of BN in that each node is independent of all its parent nodes when the value of its parent nodes are determined. Thus, the full probability formula of the BN can be expressed by the de following formula:

(2) $$P(x_{1},x_{2},\cdots ,x_{n})=\prod_{i=1}^{n}P(x_{i}|\mathrm{Parents}(x_{i}))$$

where Parents represent root nodes.

### 2.3. BN-HFACs and Failure Sensitivity

#### 2.3.1. BN-HFACs

Bayesian networks are used to quantitatively analyze human causation in HFACs, that is, BN-HFACs. Bayesian network is a good graphical tool to show the relationship between variables, and it can be used to quantitatively analyze the most likely factors causing accidents. In addition, based on the results of BN, more predictive or diagnostic suggestions could be obtained. At present, there are many studies have used BN to identify causation factors of accidents or predict the safety status under certain conditions. For example, Xia et al. [34] established BN model to predict the safety performance in the field of construction projects, while Tao et al. [37] used BN-HFACs model to analyze the human factors of air traffic accidents. Although these studies applied in different scenarios or purposes, it can be concluded that BN could be used in this paper to analyze the causes of HCAs. In addition, it must be clear is that objective and considerable data are needed in the analysis process of BN, and in this study, as many cases as possible will be collected to ensure the accuracy of the results.

Then, the implementation steps of the BN-HFACs method will be introduced. In HFACs, the factors of organization influencing are viewed as root nodes, and the unsafe behavior factors are taken as leaf nodes. The edges are connected according to the hierarchical influence of “organization influence → unsafe supervision → preconditions of unsafe behavior → unsafe behavior”, then the network topology diagram of BN, also called DAG, is formed.

Suppose there are n nodes, signed as $X_{0},X_{1},\cdots X_{n-1}$, in the network, which $X_{0}$ represent unsafe behavior, $X_{1}\cdots X_{n-1}$ represent all factors of preconditions of unsafe behavior, unsafe supervision, and organizational influence. The actual state value of the node $X_{i}(i=0,1,\cdots n-1)$ is represented by two state values, 0 and 1, which represent the non-failure state, namely, there is no unsafe behavior and there is no unsafe supervision, and failure state, namely, there is unsafe behavior, there is unsafe supervision, respectively. Therefore, the probability of occurrence of unsafe behavior can be calculated by using the joint probability distribution, as in Equation (3):

(3) $$P(X_{0}=1)=\sum_{x_{1}\cdots x_{n}}^{}P(X_{0}=1,X_{1}=x_{1},X_{2}=x_{2},\cdots ,X_{n-1}=x_{n-1})$$

In addition, BN can also be used to calculate other events’ posterior probability when some events occur. Equation (4) shows the posterior probability of other nodes when $X_{j}$ is designated as a failure state:

(4) $$P(X_{i}=1|X_{j}=1)=\frac{P(X_{j}=1|X_{i}=1)P(X_{i}=1)}{P(X_{j}=1)},0\leq i,j\leq n-1$$

#### 2.3.2. Failure Sensitivity

Sensitivity analysis is a method to study and analyze the sensitivity of state or output changes of a system (or model) to changes of system parameters or surrounding conditions; therefore, the failure sensitivity analysis can be used to determine which factors contribute more when the upper factors fail. The Bayesian network can also be used to calculate the failure sensitivity of various factors in the HFACs model [38]. In this study, failure sensitivity (FS) is defined as the change rate of the failure probability of consequence events caused by the failure state of other cause events. The formula can be expressed as Equation (5):

(5) $$FS_{ij}=\left\{ \begin{array}{rr} \frac{P(X_{j}=1|X_{i}=1)-P(X_{j}=1|X_{i}=0)}{P(X_{j}=1|X_{i}=0)}, & \Delta P\geq 0\\ 0, & \Delta P<0 \end{array}\right.,0\leq j<i\leq n-1$$

where $X_{i}$ represents cause events, the lower level factors in HFACs, $X_{j}$ represents direct consequence events, the upper-level factors in HFACs, and ΔP represents $P(X_{j}=1|X_{i}=1)-P(X_{j}=1|X_{i}=0)$.

### 2.4. Data Collection and Coding Process

#### 2.4.1. Data Collection

The investigate reports of 89 HCAs in China in recent years that were collected in this study initially. These 89 cases were screened according to the following three principles. Firstly, due to the unclear causes’ analysis of investigation reports before 2010, HCAs before 2010 were deleted. Second, large-scale accidents with the number of deaths being equal to or greater than three were selected. Thirdly, HCAs that were directly caused by human factors were selected. Based on the above three principles, 25 accidents before 2010, four accidents with less than three deaths, and 21 accidents caused directly by non-human factors were deleted. Finally, 39 cases of HCAs were collected; among them, 29 were from the official website of China Chemical Safety Association [39] and 10 were from the website of Baidu.

#### 2.4.2. Coding Process

According to the revised HFACs, the common factors of HCAs were determined. The 39 HCAs’ investigation reports were coded by corresponding to the factors of revised HFACs. Taking the poisoning and asphyxiation accident of “Tianchang Bozi Manufacturing Co., Ltd. (Chuzhou City, China) on 23 May 2008” as an example, according to the accident investigation report, the direct reason is that “operator Bi violated the relevant regulations of limited space operation and entered the limited space without wearing an air respirator and safety rope, resulting in inhaling hydrogen sulfide gas and death”. Thus, the direct cause of the accident was judged as “operation violation”. Furthermore, the indirect causes include “failure to fulfill the enterprise production safety responsibility”, “confusion of safety management”, “failure to implement safety management and operation regulations”, “formalize safety education”, “weak awareness of safety”, “low ability of self-help and mutual rescue”, “inadequate management of labor protection equipment”, “serious deficiency of on-site management”, etc. These reasons were recorded and coded; thus, direct and indirect reasons for 39 HCAs were collected.

## 3. Results

### 3.1. Transform HFACs into the Topology of BN

This section may be divided by subheadings. It should provide a concise and precise description of the experimental results, their interpretation, as well as the experimental conclusions that can be drawn.

**Figure 2:**
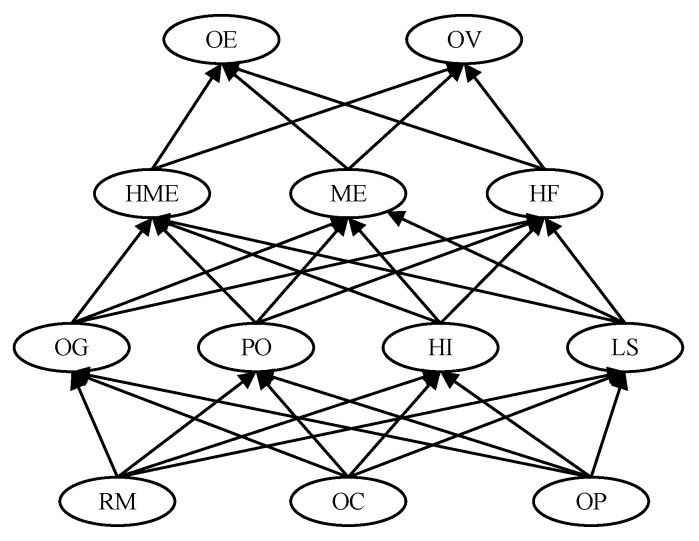
The topological structure of BN-HFACs for HCAs.

Based on the above HFACs model, a conceptual model of BN-HFACs was constructed for the analysis of human factors HCAs. As shown in Figure 2, there is a four-layer BN with unsafe behavior at the top, and, in turn, preconditions, unsafe supervision, and organizational influence one by one. According to the influence principle of layer-by-layer, the connection of BN was established. Unsafe behavior was regarded as the top event, that is, OE and OV are leaf nodes in BN. The organizational influence, unsafe supervision, and precondition of unsafe behavior were regarded as cause events of unsafe behavior, in which organizational influence is the parent node of BN, that is, RM, OC, and OP are parent nodes. This study assumed that the underlying factors affect the upper-level factors, and all the underlying factors affect the upper-level factors, thus, OG, PO, HI, LS are the parent nodes of HME, ME and HF, and are also child nodes of RM, OC, and OP. Moreover, HME, ME, and HF are parent nodes of OE and OV.

### 3.2. Probability Matrix of BN-HFACs

The above factors involved in each of the 39 cases were coded and counted, and then, the conditional probabilities of the upper factors under different states of the underlying factors were calculated. Thus, the probability matrix of 12 nodes in the network was obtained through the following processes.

The probability matrix of Level 4 was determined by the frequency of each factor that occurred in 39 cases. For example, the number of RM occurred as the cause of 39 accidents is 9, thus, the failure probability of RM was defined as 0.23, and the corresponding non-failure probability was 0.77. Similarly, the failure probabilities of OC and OP were 0.56 and 0.82, respectively, and the corresponding non-failure probabilities were 0.44 and 0.18, respectively.

For nodes in Level 3, they were affected by the different states of nodes in Level 4, thus, the probability matrix of Level 3 was determined under consideration of layer influence. For example, OG was affected by OC and OP. Under the non-failure state of OC and OP, the failure frequency of OG is 1, and the non-failure frequency is 2; thus, the failure probability of OG under this condition is 0.33, and the non-failure probability is 0.67. Furthermore, the failure probability of OG is 0.21, 0.25, and 0.5 under the state of OC non-failure OP failure, OC failure OP non-failure, and double failure of OC and OP, respectively. Moreover, if there is no corresponding state in 39 case studies, the maximum failure probability of other factors at the same level is selected as the failure probability according to the pessimistic rule. Then, the conditional probability matrix of each factor of Level 3 under the influence of Level 4 factors can be calculated, as shown in Table 2.

In a similar way, the conditional probability matrix of Level 2 and Level 1 also was obtained, as shown in Table 3 and Table 4.

### 3.3. Failure Sensitivity Analysis of BN-HFACs

**Figure 3:**
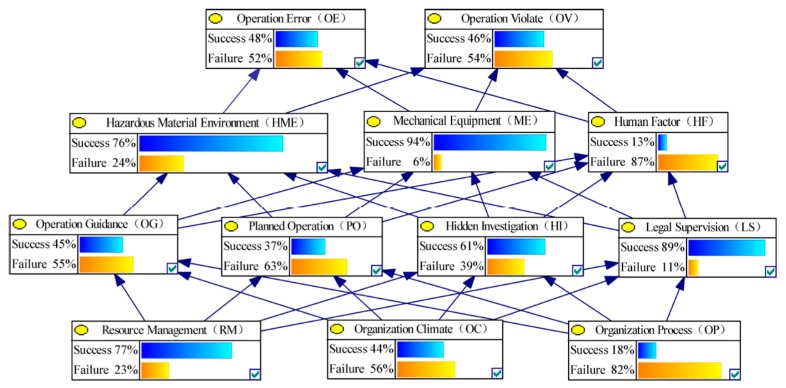
The initial BN of HFACs for HCAs.

In this section, the BN HFACS model will be established by using GeNle 2.0 software to calculate the sensitive value. First, the above probability matrix was input into the BN-HFACs model, and the initial BN can be obtained, as shown in Figure 3.

**Figure 4:**
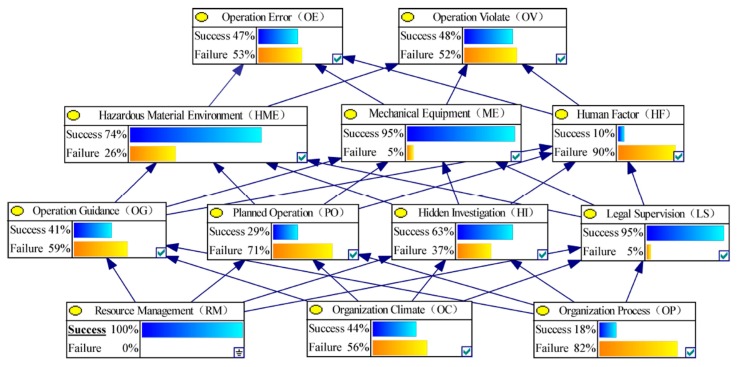
Node state probability changes when RM state is non-failure.

**Figure 5:**
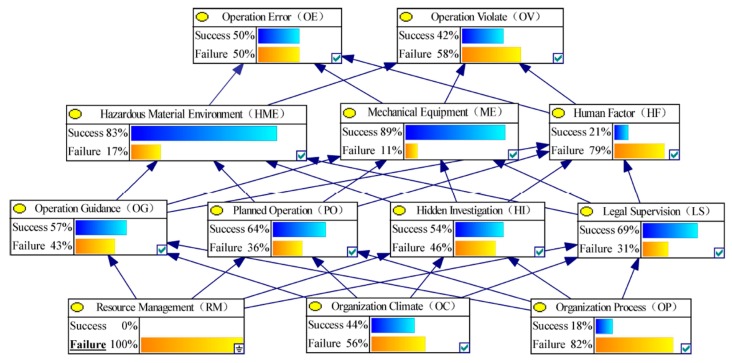
Node state probability changes when RM state is failure.

Second, the failure sensitivity of the lower node to the upper node can be calculated according to the Equation (5) by changing the state of each node in BN-HFACs. Figure 4 and Figure 5 show the non-failure or failure state or non-failure state probability change of upper nodes after controlling the RM node state. In order to explain the calculation process of failure sensitivity, the failure sensitivity calculation of OE node failure caused by RM node failure is used as an example. When RM node status is non-failure, the probability of failure state of OE is 0.53; when RM node status is failure, the probability of failure state of OE is 0.50. In this case, 0.50 is less than 0.53, the failure sensitivity is recorded as 0 according to Equation (5). Moreover, the calculation formula of the sensitivity of OV node failure caused by RM node failure can be recorded as 0.58 − 0.52/0.52 = 0.12.

Finally, the sensitivity of each upper node failure caused by the lower node failure can be calculated according to the above steps, as shown in Table 5.

Results of sensitivity analysis showed that HME and ME in Level 2 have the same direction failure effect on OE, and the sensitivity is 1.63 and 0.49, while all three elements in Level 2 do not have the same direction failure effect on OV. In addition, for factors in Level 3 and Level 4, OC, OP, OG, PO, and LS also have obvious co-invalidation effects on OE, while RM, HI, and LS have obvious co-invalidation effects on OV.

For the factors of preconditions of unsafe behavior, OG and PO in Level 3 have the same failure effect on HME, and the sensitivity is 6.60 and 3.71, respectively. HI and LS have the same failure effect on ME, and the sensitivity is 1.25 and 2.4, respectively. OG, PO, and LS in level 3 have the same failure effect on HF, and the sensitivity is 0.22, 0.21, and 0.03, respectively. In addition, for factors in Level 4, OC and OP have obvious co-effects on HME, RM has obvious effects on ME, and OC also has obvious effects on HF.

For the factors of unsafe supervision, only OC in Level 4 has the same failure effect on OG, and the sensitivity is 4.73. OC and OP have the same failure effect on PO, and the sensitivity is 1.81 and 0.1, respectively. While RM has a significant impact on HI with a sensitivity of 0.24, RM and OC have the same impact on LS, with a sensitivity of 5.2 and 4.67, respectively.

## 4. Discussion

This study established a BN-HFACs model to identify the important factors leading to unsafe behaviors in hazardous chemical accidents. At first, 12 factors that may cause accidents were divided into four levels. Then, the influence relationship among factors was shown in the form of a Bayesian network. Finally, the sensitivity analysis of a Bayesian network is used to calculate the failure sensitivity of the influencing factors among different levels based on 39 HCAs.

### 4.1. Theoretical Implication

Firstly, hazardous material environment and mechanical equipment are the direct cause of the operational error. Combining with the research background of this paper, it could be explained as follows, when chemical gas leakage, abnormal chemical reaction, or bad meteorological conditions occur in the workshop, or the state of production and storage equipment is unsafe, operators are prone to operate errors. This finding corresponds with other relevant research. In the study of railway accidents by Madigan et al. [40], the operating environment was confirmed as an important factor that leads to the driver’s operational errors. In detail, the unconventional operating environment could distract the driver’s attention and lead to accidents. Environmental factors also proved to be an important cause of unsafe behaviors in the research regarding mine accidents by Mirzaei et al. [33]. Another study on unsafe behaviors of coal miners, although denying the direct influence of the preconditions of unsafe behaviors on unsafe behaviors, proved that the preconditions of unsafe behaviors are important intermediate variables that affect unsafe behaviors [41]. Although the above research is not in the field of hazardous chemical research, their research conclusions still support the research conclusions of this study. In addition, it may be possible to explain this finding from the perspective of risk perception. When workers are in an unconventional environment of dangerous chemicals or in the face of abnormal mechanical equipment, they often have fear psychology, which can easily cause unsafe behavior among workers [42].

Secondly, some factors in organization influence and unsafe supervision, such as organization climate, organization process, operation guidance, planned operation, and legal supervision, are also important reasons for operational errors. Scholars have confirmed that organizational factors and unsafe supervision are important factors affecting unsafe behaviors. Mirzaei et al. [43] confirmed in the study on the impact of human and organizational defects on coal miners that organizational defects have a direct positive impact on operational errors and violations, and the impact on operational errors is greater than that on operational violations. Research by Fiedlander and Evans about steam power [44] also confirmed the above finding. In addition, in this study, resource management, hidden investigation, and legal supervision have an impact on operation violates. By comparing the influencing factors of operation error and operation violation, it can be found that the supervision or organizational factors that are more related to safety have a greater influence on operation violation, such as hidden investigation and legal supervision. The above findings indicate that management’s attention to safety issues or supervision will directly affect workers’ safety awareness, resulting in violations.

Thirdly, there are still close relationships between other hierarchical elements, including the impact of operation guidance and planned operation on hazardous material environment; the impact of hidden investigation and legal supervision on mechanical equipment; the impact of resource on legal supervision; the impact of organization climate on operation guidance, planned operation, and legal supervision. These research findings indicate that there are strong interrelationships between the preconditions of unsafe behavior, unsafe supervision, and organizational factors, which also proves the systematic characteristics of HFACs. As confirmed by other studies [45], the conclusions of this study confirmed the possible ways to reduce the occurrence of hazardous chemical accidents in terms of organization, supervision, and working environment.

In fact, few scholars have considered the different influencing factors of operation errors and operation violations in empirical research. This study found the differences among the organization factors, supervision factors, and precondition of unsafe behavior in causing operation errors and operation violations. Moreover, it can be found that at the level of organizational factors, the organizational climate has the most obvious impact on other factors, indicating the importance of organizational safety culture in alleviating unsafe behavior in HCAs. This finding was also confirmed by some scholars in the field of aircraft maintenance [46], construction [47], and so on.

### 4.2. Practical Implication

Based on the above findings, suggestions are put forward from the perspectives of individuals, enterprises, and regulators to reduce the occurrence of hazardous chemical accidents.

Firstly, it is necessary to improve the occupational requirements of employees engaged in hazardous chemical plants, especially the ability of employees to cope with special hazardous situations. Of course, this requires certain measures and systems to enhance the enthusiasm of employees to improve their own abilities. For example, the enterprises encourage employees to actively participate in the hazardous chemicals skill competition and formulate reward rules; regularly invites experts to conduct education and training for employees; analyze possible hazardous scenes, and enhance employees’ familiarity with hazardous scenes.

Secondly, the enterprises of hazardous chemicals should improve in three aspects: controlling the precondition of unsafe behavior, strengthening the supervision of unsafe behavior, and enhancing the organization’s attention to safety management. In terms of controlling the precondition of unsafe behavior, the enterprises of the hazardous chemical should strengthen the condition inspection of equipment and facilities in the factory, paying close attention to the changes of the external environment. In addition, they should adopt modern technology as far as possible, and use intelligent robots instead of people to deal with the dangerous environment. From the perspective of unsafe behavior supervision, it is particularly important to formulate perfect operation guidelines and detailed operation plans to prevent the occurrence of a dangerous environment. In addition, the enterprises should strengthen the investigation of hidden dangers, correct the problems of equipment and facilities, and strengthen legal supervision to strictly control employees’ violations. Moreover, the enterprises using the hazardous chemical should pay special attention to the construction of safety culture, increase safety investment, and form a smooth operation mechanism of safety management.

Thirdly, the government supervision department should strengthen the supervision of hazardous chemical enterprises to undertake the training obligation for employees of hazardous chemical enterprises. HFACs provide a model for the government to regulate hazardous chemicals enterprises. HFACS can not only be used for accident investigation after the accidents occur, but can also be used as a standard for the daily supervision of hazardous chemical enterprises. In addition, government regulatory authorities should also issue more support policies to encourage enterprises with outstanding safety work. Moreover, governmental departments can organize skill competition with hazardous chemical enterprises to provide opportunities for employees to improve their hazardous chemicals skills.

### 4.3. Limitations and Future Research Directions

Although the above theoretical and practical implications were obtained by this study, there still exist several limitations. First, BN-HFACs is a good method of accident cause analysis, but it needs a lot of data to ensure the preciseness of the result. In the present study, only 39 HCA cases were selected and the pessimistic principle was used to determine the probability in the non-existent state when calculating the probability matrix, which may affect the stability of the results. Second, the expert experience is a good basis for the analysis of accident causes, but the research on hazardous chemicals experts is not carried out in this study. Therefore, in future research, the case database and expert experience should be gradually enriched to verify the accuracy of the research results, or new models should be developed to produce more reasonable results with a small number of samples. In addition, research on the cause of dangerous chemical accidents in other countries of the world should also be carried out.

## 5. Conclusions

Hazardous chemical accidents have a great impact on public life, property, and health. Reducing the probability of hazardous chemical accidents is of great significance to improve the public sense of safety and happiness. In this present study, HFACs for HCAs were proposed according to the characteristics of hazardous chemical accidents and hazardous chemical safety management. The method of BN-HFACs was successfully used to analyze the factors that lead to human unsafe behavior in HCAs. In total, 39 investigation reports of HCAs were collected and used to build BN-HFACs. Based on the results of failure sensitivity analysis, the following conclusions have been obtained. First, hazardous material environment (1.63) and mechanical equipment (0.49) are the direct cause of the operational error. Second, some factors in organization influence and unsafe supervision, such as organization climate (0.34), organization process (0.08), operation guidance (0.37), planned operation (0.22), and legal supervision (0.19), are important reasons for operational errors. Third, resource management (0.12), hidden investigation (0.18), and legal supervision (0.13) have an impact on operation violates. Fourth, there are still close relationships between other hierarchical elements. Fifth, the organizational climate has the most obvious impact on other factors at the level of organizational factors. In view of the above conclusions, the corresponding policy suggestions were put forward from the perspective of individual, enterprise and government. In future research, HCA cases will continue to be enriched and expert experience will be absorbed, and the research on the causes of hazardous chemical accidents will be extended worldwide.

## Figures and Tables

**Table 1:** Explanation of subdivision indexes of HFACs for HCAs.

Level	Subdivision Index	Explanation of Subdivision Index
UB	OE	OE refers to the operator’s unintentional wrong behavior
	OV	OV refers to the operator’s non-compliance with the established plant regulations
PUB	HME	HME means environmental factors related to hazardous chemicals, and there are three kinds of environmental factors including chemical gas leakage, chemical reactions, and meteorological conditions
	ME	ME refers to the safety state of equipment with the functions of production and storage
	HF	HF includes risk identification ability, safety awareness, safety skills, and mutual rescue consciousness
US	OG	OG includes three aspects, they are safety instruction from managers to operators, the supervision of managers about confined space, the supervision of managers to urge the operators to comply with the regulations
	PO	PO mainly involves the operability of the operating procedures
	HI	HI includes two aspects, one is the risk perception of managers, and the other is that the managers do not eliminate the known hidden dangers timely
	LS	LS divides two facets, including operating tickets’ management conforms to regulation and managers obey the rules and regulations
OI	RM	RM mainly refers to the input of safety production
	OC	OC includes three aspects, they are enterprise safety responsibility, enterprise awareness of production safety, enterprise rules, and regulations about safety
	OP	OP includes safety management, safety education and training, emergency management, third-party evaluation, and so on

**Table 2:** Conditional probability matrix of Level 3.

**Nodes Status**	**RM-S**				**RM-F**			
	**OC-S**		**OC-F**		**OC-S**		**OC-F**	
	**OP-S**	**OP-F**	**OP-S**	**OP-F**	OP-S *	**OP-F**	**OP-S**	**OP-F**
OG-S	0.67	0.70	1.00	0.50	0.00	1.00	0.00	0.50
OG-F	0.33	0.30	0.00	0.50	1.00	0.00	1.00	0.50
PO-S	0.67	1.00	0.67	0.71	0.00	0.75	0.00	0.50
PO-F	0.33	0.00	0.33	0.29	1.00	0.25	1.00	0.50
HI-S	0.67	0.50	0.67	0.71	0.50	0.75	0.00	0.50
HI-F	0.33	0.50	0.33	0.29	0.50	0.25	1.00	0.50
LS-S	1.00	0.90	1.00	0.86	0.50	0.75	1.00	0.50
LS-F	0.00	0.10	0.00	0.14	0.50	0.25	0.00	0.50

Notes: S stands for the non-failure state, F stands for failure state, * represents no corresponding state.

**Table 3:** Conditional probability matrix of Level 2.

Nodes Status	OG-S							
	PO-S				PO-F			
	HI-S		HI-F		HI-S		HI-F	
	LS-S	LS-F *	LS-S	LS-F	LS-S	LS-F	LS-S	LS-F
HME-S	0.93	0.50	0.67	1.00	1.00	1.00	1.00	1.00
HME-F	0.07	0.50	0.33	0.00	0.00	0.00	0.00	0.00
ME-S	0.86	0.67	1.00	0.67	1.00	1.00	1.00	1.00
ME-F	0.14	0.33	0.00	0.33	0.00	0.00	0.00	0.00
HF-S	0.36	0.00	0.00	0.67	0.00	0.00	0.50	0.00
HF-F	0.64	1.00	1.00	0.33	1.00	1.00	0.50	1.00
**Nodes Status**	**OG-F**							
	**PO-S**				**PO-F**			
	**HI-S**		**HI-F**		**HI-S**		**HI-F**	
	**LS-S**	LS-F *	**LS-S**	**LS-F**	**LS-S**	LS-F *	**LS-S**	LS-F *
HME-S	1.00	0.50	0.50	1.00	1.00	0.50	0.67	0.50
HME-F	0.00	0.50	0.50	0.00	0.00	0.50	0.33	0.50
ME-S	0.83	0.67	1.00	1.00	1.00	0.67	1.00	0.67
ME-F	0.17	0.33	0.00	0.00	0.00	0.33	0.00	0.33
HF-S	0.33	0.00	0.50	0.00	0.00	0.00	0.67	0.00
HF-F	0.67	1.00	0.50	1.00	1.00	1.00	0.33	1.00

Notes: S stands for the non-failure state, F stands for failure state, * represents no corresponding state.

**Table 4:** Conditional probability matrix of Level 1.

Nodes Status	HME-S				HME-F			
	ME-S		ME-F		HME-S		HME-F	
	HF-S	HF-F	HF-S *	HF-F	HF-S	HF-F	HF-S	HF-F
OE-S	0.50	0.68	0.00	0.33	0.00	0.00	1.00	0.00 *
OE-F	0.50	0.32	1.00	0.67	1.00	1.00	0.00	1.00 *
OV-S	0.40	0.27	0.00	0.67	1.00	1.00	0.00	0.00 *
OV-F	0.60	0.73	1.00	0.33	0.00	0.00	1.00	1.00 *

Notes: S stands for the non-failure state, F stands for failure state, * represents no corresponding state.

**Table 5:** Failure sensitivity of nodes in BN-HFACs.

	RM	OC	OP	OG	PO	HI	LS	HME	ME	HF
OG	0.00	4.73	0.00	—	—	—	—	—	—	—
PO	0.00	1.81	0.10	—	—	—	—	—	—	—
HI	0.24	0.00	0.00	—	—	—	—	—	—	—
LS	5.20	4.67	0.00	—	—	—	—	—	—	—
HME	0.00	4.29	0.39	6.60	3.71	0.00	0.00	—	—	—
ME	1.20	0.00	0.00	0.00	0.00	1.25	2.4	—	—	—
HF	0.00	0.25	0.01	0.22	0.21	0.00	0.03	—	—	—
OE	0.00	0.34	0.08	0.37	0.22	0.00	0.19	1.63	0.49	0.00
OV	0.12	0.00	0.00	0.00	0.00	0.18	0.13	0.00	0.00	0.00

Note: columns indicate cause events, rows indicate result events.
